# Trends of Anger and Physical Aggression in Russian Women During COVID-19 Lockdown

**DOI:** 10.3389/fgwh.2021.698151

**Published:** 2021-10-20

**Authors:** Alexandra Klimovich-Mickael, Norwin Kubick, Elena Milanesi, Maria Dobre, Marzena Łazarczyk, Baraba Wijas, Mariusz Sacharczuk, Michel-Edwar Mickael

**Affiliations:** ^1^PM Forskningscenter, Stockholm, Sweden; ^2^Institute of Biochemistry, Molecular Cell Biology, University Clinic Hamburg-Eppendorf, Hamburg, Germany; ^3^Victor Babes National Institute of Pathology, Bucharest, Romania; ^4^Institute of Genetics and Animal Biotechnology of the Polish Academy of Sciences, Magdalenka, Poland

**Keywords:** angriness, anxiety, depression, coronavirus, psychological impact

## Abstract

The effect of social lockdown during the COVID-19 outbreak on female aggressiveness is not well known. The strict measures of lockdown have resulted in millions of people, worldwide, confined to their homes during the pandemic. However, the consequence of lockdown strategies on females' psychological status including aggressiveness has not yet been investigated. We conducted a cross-sectional study on 31 Russian females' homemakers who are participants in an online fitness platform to investigate the immediate anxiety, depression, and aggression experienced under strict lockdown measures. The participants were surveyed using the hospital anxiety depression scale (HADS) and the Buss-Perry Aggression Questionnaire. We used descriptive and statistical methods to investigate the prevalence of these emotions among two age groups (20–35 and 36–65 years). We found that moderate anxiety prevalence was 77.4% in the entire group while mild depression was 54.8%. Interestingly, the whole sample showed a high level of angriness (*p* = 0.0002) and physical aggression (*p* = 0.019). These two emotions seem to be more prevalent than other negative emotions such as hostility, verbal aggression. This relationship was not dependent on age. Overall, there is a significant worsening in female aggression that could lead to higher chances of female victimization and being subjected to partner violence. Future policies designing lockdown strategies should consider this effect on active female homemakers. Due to the small size of our cohort, our results are only indicative of data trends. Larger studies are still needed to confirm the current findings.

## Introduction

Aggression among couples can take various forms that are united by the intention to cause harm to another person. One classification of aggression activities is by their effect, where aggressive behavior is indicated as a physical or verbal action. Physical aggression constitutes performing actions of corporal nature such as hitting or slapping. Verbal aggression, on the other hand, is manifested by various negative verbal communication behaviors such as shouting. Aggression could also be categorized by its nature of interaction into two main categories (i) direct and (ii) indirect. Direct aggression includes confrontation, while indirect aggression constitutes other non-confrontational actions such as spreading false rumors. Aggression could likewise take the form of an impulsive action, which is normally initiated by anger. It can similarly manifest as a reaction to hostile action and it is defined as being reactive (1). Interestingly, analysis of past surveys, showed that an equal percentage of men and women report using physical aggression between couples (2). In some instances, the percentage of women who reported performing physical aggression was higher than men (3). The American National Family Violence Survey analyzed the responses of 6,002 participants (2) and the results indicated that in the year prior to the survey at least 12.4% of women reported that they perpetuated violent acts toward their husbands, while only 11.6% of the husbands admitted engaging in physical aggression toward their wives. This has similarly been mirrored in severe violence investigations, where the self-reporting percentage of women who committed severe violent acts was 4.8% while for men it was 3.4%. These results were correspondingly supported by analysis of self-reporting surveys of college students addressing physical aggression (4). In Russia, the statistics mirror international trends in time pre-pandemic, with males reported utilizing physical aggression scores significantly higher than females, while females slightly exceeding males in using verbal aggression, anger, and hostility up, with a mean between 12 and 24% (5, 6). These differences in aggression behavior are similar in both school children as well as adult Russians (7).

In the course of the COVID-19 outbreak, a significant upsurge in aggressive domestic violence toward women has been reported (8). However, the reasons behind this phenomenon are not yet known. Up to date, few reports attempted to decipher aggressive behavior under lockdown and how it is affecting women. World Health Organization reported that there have been 400,435 confirmed cases of COVID-19 with 3,620 deaths in May 2020 in the Russian Federation. To fight the spread of COVID-19 in Russia, various governmental restrictive measures have been implemented, such as limiting people's movements (9). These restrictions included social distancing, the closing of gyms and parks, travel restrictions, quarantines, as well as stay-at-home guidance (4, 9). The associated stress of prolonged uncertainty caused by home confinement has been reported to affect mental well-being (10, 11). Stress and anxiety were reported to significantly increase among healthcare workers during the COVID-19 pandemic in Russia (12). Additionally, a relationship between excessive consumption of media news about COVID-19 and increased anxiety in Russia has also been reported (13). An association between depression symptoms caused by COVID-19 and physical activity in Germany, Italy, Spain, and Russia has also been reported (14). Several reports demonstrated that in previous epidemics the occurrence of stress-associated symptoms including post-traumatic stress disorder, anxiety, depression, anger, and female victimization has increased (15–17). However, if female aggression increases during epidemics lockdown is not yet known. Also, the psychological factors leading to female aggression during lockdown have been not yet investigated. We hypothesized that the lockdown strict strategy can augment aggressive behavior through invoking negative emotions such as anxiety, depressive-like symptoms, angriness, and hostility. This in turn could lead to physical aggression by women toward their spouses.

The main questions, this report addresses are whether there is a rise in women's physical aggression due to the pandemic lockdown policies and whether this rise is correlated to an increase in anxiety and depression. We performed an exploratory cross-sectional study to investigate trends of anxiety, depression, and aggression among females under lockdown conditions. We recruited 31 Russian homemakers' women participating in a fitness program online. Our survey measured anxiety, depression, verbal aggression, physical aggression, and anger. Our results suggest that there might be a correlation between the rise in anxiety, angriness feelings, and physical aggression during strict lockdown guidance.

## Methods

### Study Design

A cross-sectional study was designed to measure anxiety, depression, and aggression experienced by homemakers, attending an online fitness program under strict lockdown measures in Russia. To this aim, we conducted an anonymous non-interventional online survey using the hospital anxiety depression scale (HADS) and the Buss-Perry Aggression Questionnaire. The surveys were completed in May 2020 under lockdown conditions, where individuals were recommended to stay indoors except for emergencies and purchasing of food.

### Procedure and Sample

Sample size calculations were performed using Power = 1–β.= 0.85 and α = 0.1. The traditional sample size calculations are based on 5% for alpha and 80% for power level. We used a power level of 85% to ensure taking into consideration a possible false-negative high rate, whereas we tolerated a higher “false positive rate,” because of the risk of not finding enough participants due to lockdown conditions. The correlation coefficient under the alternative hypothesis was 0.40, while for the null hypothesis it was 0.1. We used MedCal© and G^*^power 3.1.9.7 to calculate the sample size. The estimated sample size is 71. Coefficient estimation is based on previous reports and our own experience. Because of the relatively low number of participants, our results are only indicators of the trends of increased aggression by women under strict lockdown regulations. However, the danger posed by the strict lockdown conditions on women's victimization highlights the immediate need of investigating the causes of this phenomenon further.

The study included 31 female homemakers, who participated in a fitness platform https://vk.com/marafonlovelyme. We selected homemakers for this program to eliminate the possibility that aggressive behavior is due to loss of work because of the pandemic, as there seems to be a direct link between loss of work and aggressive behavior in men and women (18). Furthermore, there seems to be no difference in the stress level between homemakers and working women in pre-pandemic reports (19). However, homemakers are more likely to suppress their anger (20, 21). We chose the sample from a fitness group as exercise is reported to reduce aggressive behavior (22). Also, the sample was readily available through online access for our researchers to conduct the surveys. We contacted the organizers of the online fitness platform *Marafonlovelyme* to have access to Russian females participating in their fitness programs. After the organizers provided us with their consent, the link to the survey was sent together with a form detailing the instructions. The questionnaire answers were collected anonymously from the consented participants. Data were stored in excel files and analyzed by SPPS. The participants reported age ranged from 20 to 65 years. The education level of participants varied from lower secondary education to master's degree. The main purpose of our study was to examine if lockdown policy has caused depression, anxiety and in turn if these two emotions caused a rise in aggressive behavior among women. Thus, we excluded participants who were already suffering from known clinically diagnosed depression before the pandemic. The participants were categorized into two age groups: 20–35 years (*N* = 13) and 36–65 years (*N* = 18). The age stratification was performed according to the literature (23). As at the time of conducting the survey all Russian territories were subjected to strict lockdown, we had to employ a standard quasi-experimental evaluation design.

### Measuring Instrument and Statistical Analysis

#### Depression and Anxiety Measurement

We employed the hospital anxiety depression scale (HADS). The HADS tool has been used widely in medical situations and investigation reports (24). This tool is valid for identifying anxiety disorders and symptoms severity of anxiety and depression in hospital practice primary care as well as the general population. It is comprised of two statements focusing on either anxiety or depression. The statements created to measure the depressive component of the scale are designed to pinpoint if there is a decrease in the ability to experience pleasure, such as “I have lost interest in my appearance.” While statements that assess anxiety level use a form similar to “I feel tense or wound up.” The whole scale consists of seven statements relating to anxiety as well as seven relating to depression. The scoring system utilized a 4-point response category (0–3), such that the possible scores range is from 0 to 21. The Depression and Anxiety subscale scores were calculated by adding the numbers for both anxiety and depression distinctly. The interpretation of HADS scores is normal (0–7), mild (8–10), moderate (11–14), and severe (15–21). The HADS questionnaire took between 5 and 10 min to complete, and an explanation was provided to explain the purpose of the questionnaire. The HADS has been translated by the MAPI Research Institute into the Russian language and has been previously validated (25).

#### Aggression Measurement

Aggression was measured by Buss-Perry Aggression Questionnaire (BPAQ) (26). This scale consists of four subscales of physical aggression (PA), verbal aggression (VA), anger (Ang), and hostility (Host). The partakers were requested to estimate each item utilizing a 5-point Likert scale ranging from 1 (not true) to 5 (true) (27). The descriptive scores of the four subscales were considered by averaging the item scores and scoring was done as described earlier (26). The BPAQ was translated to Russian and validated by Yenikolopov and Tsibulsky in 2007 (28). All statistical analyses were performed using the 17.00 SPSS program. For the Buss-Perry Aggression Questionnaire, internal consistency of the scales was calculated with Cronbach's alpha coefficient. The non-parametric Mann-Whitney test was used to compare means of the BPAQ total score, BPAQ subcategories (physical aggression, verbal aggression, angriness, and hostility) scores, and HADS scores between the two age categories (20–35 vs. 36–65 years). The correlations among scores of BPAQ subcategories have been calculated using Pearson correlation test. A single *t*-test (also known as one-sample *t*-test) was used to compare the mean scores of BPAQ subcategories and total scores between our cohort and the average scores obtained by Buss and Perry. The One Sample *t*-test examines whether the mean of a population (results of our study group) is statistically different from a known or hypothesized value (e.g., Buss and Perry data).

### Study Protocol and Ethical Considerations

The study protocol was as follows; we contacted a fitness platform that is focused on Russian women. The fitness platform consented to share the link on the platform's news feed. Women volunteered anonymously for filling up the survey in the google survey platform after filling in the consent form. Only women without any known clinically diagnosed depression were recruited. Since the survey was anonymous and no identifiable personal data or IP addresses were collected, ethical approval was not mandatory due to the non-interventional online survey research design. However, ethical approval was obtained by the 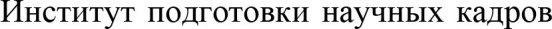 HAH 
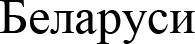
 (Institute of Scientific Personnel Training of the National Academy of Sciences).

## Results

This study was designed to measure anxiety, depression, and aggression experienced by females during the peak of the COVID-19 epidemic under strict lockdown measures. This data has the perspective to reveal the alterations in aggressiveness in women during the pandemic and aids in advancing novel psychosocial precautions for future epidemic handling. Our main findings indicate that Russian women under lockdown reported feeling of moderate level of anxiety, mild level of depression as well as hostility and verbal aggression. However, they reported significant levels of angriness and physical aggression.

### HADS: Anxiety and Depression

The internal consistency of HADS was calculated by Cronbach's α. Cronbach's alpha was 0.865 and 0.833 for anxiety and depression, respectively. Our investigation revealed that 77.4% of women under lockdown reported symptoms of moderate anxiety and the frequency did not differ between the two age groups. The scores of the moderate anxiety were not statistically different between the two age groups as well (20–35 years = 12.70 ± 1.25, 36–65 years = 12.36 ± 1.15, *p* = 0.508). Regarding depression, mild depressive symptoms were detected in 54.8% of the entire cohort, with a higher frequency in the younger women group (76.9%) compared to the 36–65 age group (38.9%) without reaching statistical significance when considering the three categories of depression (normal, mild, moderate) in the two age categories (χ^2^ = 4.409, *p* = 0.089). Moreover, only two women with moderate depression (36–65 age group) were found in our cohort and no cases with severe depressive symptoms were identified (Table 1).

**Table 1:** Results of the HADS test.

	**HADS score**	**Entire cohort (***N*** = 31)**		**Age 20–35 (***N*** = 13)**		**Age 36–65 (***N*** = 18)**	
		**N**	**Percentage**	**N**	**Percentage**	**N**	**Percentage**
Anxiety	Normal (0–7)	2	6.5%	1	7.7%	1	5.5%
	Mild (8–10)	4	12.9%	2	15.4%	2	11.1%
	Moderate (11–14)	24	77.4%	10	76.9%	14	77.8%
	Severe (15–21)	1	3.2%	0	0%	1	5.6%
Depression	Normal (0–7)	12	38.7%	3	23.1%	9	50.0%
	Mild (8–10)	17	54.8%	10	76.9%	7	38.9%
	Moderate (11–14)	2	6.5%	0	0%	2	11.1%
	Severe (15–21)	0	0%	0	0%	0	0%

### Aggressive Test Results

**Figure 1:**
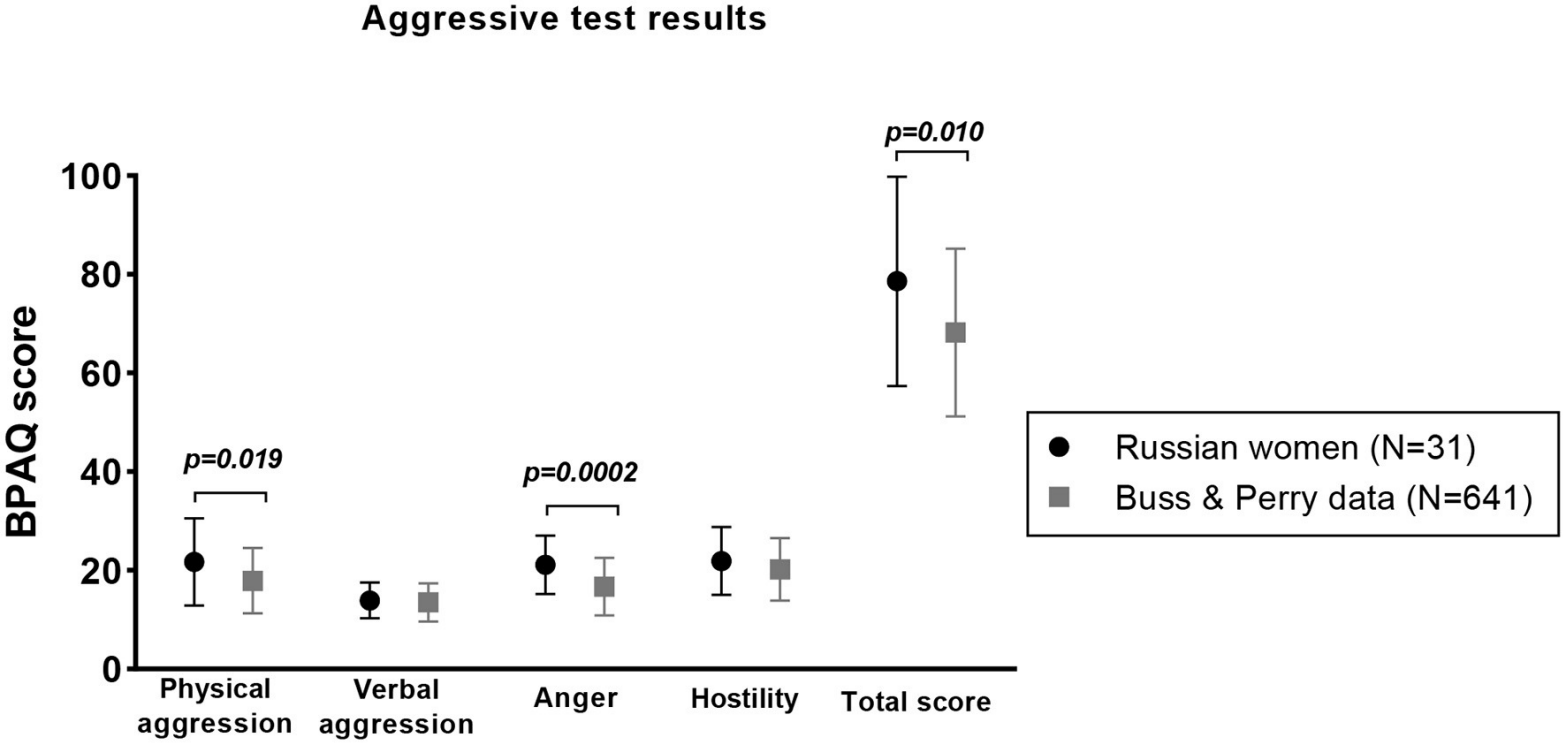
Mean value ± SD of BPAQ score in Russian women compared to Buss and Perry values.

Regarding the reliability, the internal consistency of the Buss Perry test was evaluated utilizing Cronbach's alpha (α). Values of α between 0.70 and 0.80 represent good reliability estimates (29), however, a lower value (around 0.60) on scales is acceptable (11, 30). In our study, α values were as follows: 0.855 for physical aggression, 0.663 for verbal aggression, 0.715 for anger, and 0.821 for hostility. Comparing the mean values obtained in our women cohort with those reported by Buss and Perry in 1992 on a cohort of 641 women, we found that the investigated Russian women cohort showed significant high score for physical aggression (*p* = 0.019) and anger (*p* = 0.0002) as well as total score (*p* = 0.010; Figure 1 and Table 2). We finally performed a logistic regression analysis in order to find associations between Buss and Perry's aggression score, anxiety, and depressive symptoms, as well as with age category. We found a significant relationship between anxiety and aggression score (*p* = 0.022).

**Table 2:** Results of the Buss-Perry test.

	**Entire cohort (***N*** = 31)**				**Women score ***N*** = 641 (Buss and Perry, 1992)**	**Significance**
Buss-Perry scale	Cronbach α	Mean (SD); (SE)	Min-Max	Variance	Mean (SD)	*p*-value (Single *t*-test)
Physical aggression	0.855	21.7 (8.8); (1.5)	9–45	76.9	17.9 (6.6)	*p* = 0.019 (*t* = 2.458)
Verbal aggression	0.663	13.9 (3.6); (0.6)	8–21	12.8	13.5 (3.9)	*p* = 0.536 (*t* = 0.625)
Anger	0.715	21.1 (5.9); (1.1)	12–31	34.4	16.7 (5.8)	*p* = 0.0002 (*t* = 4.139)
Hostility	0.821	21.9 (6.9); (1.2)	12–39	48.1	20.2 (6.3)	*p* = 0.189 (*t* = 1.341)
Total score	0.919	78.6 (21.2); (3.8)	47–133	450.6	68.2 (17.0)	*p* = 0.010 (*t* = 2.731)

In particular, a score above the Buss Perry mean for physical aggression and anger was reported for 21 women (67.7%) and 24 women (77.4%), respectively. A total score above the mean was observed in 20 cases (64.5%). The correlations among the scores of the Buss Perry subcategories are reported in Table 3. We further compared the Buss Perry scores in the two age categories and we did not find any statistical difference between the two groups (Table 4).

**Table 3:** Correlations between scores of the Buss Perry subcategories.

	**Physical aggression**	**Verbal aggression**	**Anger**	**Hostility**	**Total score**
	**(95% CI)**	**(95% CI)**	**(95% CI)**	**(95% CI)**	
Physical aggression (BPQ)	//				
Verbal aggression (BPQ)	0.666** (0.408–0.826)	//			
Anger (BPQ)	0.587** (0.294–0.779)	0.748** (0.535–0.871)	//		
Hostility (BPQ)	0.541** (0.231–0.752)	0.544** (0.235–0.753)	0.630** (0.355–0.805)	//	
Total score (BPQ)	0.865** (0.736–0.933)	0.829** (0.672–0.915)	0.851** (0.711–0.926)	0.817** (0.651–0.908)	//

** *p < 0.01*.

**Table 4:** Buss Perry scores in our cohort categorized by age.

	**Age 20–35 (***N*** = 13)**	**Age 36–65 (***N*** = 18)**	***p*** **-value**
Buss-Perry scale	Mean (SD)	Mean (SD)	
Physical aggression	19.5 (6.9)	23.4 (9.8)	0.312
Verbal aggression	13.2 (3.2)	14.4 (3.9)	0.489
Anger	19.3 (5.5)	22.3 (6.0)	0.135
Hostility	21.7 (8.1)	22.0 (6.2)	0.737
Total score	73.8 (20.3)	82.1 (21.7)	0.332

## Discussion

This study was designed to explore the immediate anxiety, depression, and aggression experienced by female participants without known psychiatric illnesses during the peak of the COVID-19 epidemic under strict lockdown measures. We hypothesized that lockdown affected the mood of females in an age dependent manner, leading to the onset of depression, anxiety symptoms and contributed to physical aggression committed by women.

The data in this study suggested that Russian females' levels of anxiety and depression increased during COVID-19 lock-down. Previous reports indicated that there is a higher risk of elevated symptoms of anxiety, depression, and sleep disorders during the lockdown. Similar to previous reports, our data indicated that 77.4% of women under lockdown reported symptoms of moderate anxiety, while 54.8% of the entire cohort reported symptoms of mild depression (15). It is important to note that the values for these two categories of symptoms under normal conditions among Russian women were 4 and 25%, respectively (31). We did not find a significant difference between the two age groups studied. This may indicate that the rise in depression and anxiety levels under lockdown is not age dependent. However, partially in line with Huang and Zhao, we observed a trend indicating that younger participants (<35 years) were more likely to develop mild depressive symptoms during the COVID-19 outbreak than older participants (>36 years) (32).

Angriness was self-reported by most of the participants, regardless of their age group. The percentage of the reported feeling of anger was 77.4%, compared to 12–24% pre-pandemic (6). Anger was reported to be a common feeling in various countries such as Korea and Spain during the COVID-19 lockdown (33, 34). Anger could be accompanied by other negative emotions such as emotional disturbance, depression, stress, low mood, irritability, insomnia, post-traumatic stress symptoms, emotional exhaustion (12, 17). These negative emotions could be contributing to increasing angriness levels. Also, one of the reasons that could lead to feeling angry could be related to the feeling of loss (35). During strict lockdown conditions, individuals are subjected to loss of time, sense of normality, in-person contacts, and financial resources. The feeling of loss could be causing a provocation reaction and thus ultimately increasing anger (36). Interestingly, there is no significant difference between the two age groups reported angriness. These findings indicate that anger does not seem to be age dependent.

Feelings of anxiety and angriness could be one of the primary causes leading to physical aggression. A rise in symptoms related to anxiety, depression, and sleep disorders during the COVID-19 pandemic, has been reported (37). Aggression behavior includes hostility, verbal aggression, and physical aggression. Hostility between couples can cause an increase of 81 and 39% in cortisol and prolactin levels, respectively, in women compared to their husbands (38). Higher cortisol and prolactin levels are associated with anxiety (39). Thus, anxiety could be correlated to a rise in hostility. However, our analysis did not show any significant levels of hostile emotions (Table 2). Females could inflict verbal aggression associated with minor physical aggression (40). It was suggested that verbal aggression is an alternative to physical aggression implying that verbal and physical aggression could manifest the same underlying phenomenon (41). Conversely, marital aggression was suggested to be a two-step process that starts with verbal aggression and escalates to physical aggression (41). Our investigation did not identify verbal aggression in the sample studied (Table 2). Conversely, we found a significant increase in anxiety, angriness, and physical aggression during the lockdown and a mild increase in depression (Tables 1, 2). Taken together, our findings support the assumption that verbal aggression constitutes an alternative activity to physical aggression, and that physical aggression is correlated to the feeling of anxiety.

One of the critical risks of female physical aggression is increasing female victimization (42). Although men and women may experience interpersonal violence, women are more likely to experience sustained forms of emotional, psychological, or physical abuse culminating in injury or death. Women's use of violence is mostly related to their ongoing victimization, using force to stop or escape violence (43). However, there has been an increase in the global domestic violence offense committed by females (44). In the course of COVID-19 outbreak, an alarming increase in aggressive domestic violence toward women has been reported. This phenomenon echoes earlier reports described during previous pandemics and epidemics (45). Our data indicate a high prevalence of self-reported angriness and physical aggression (Table 2). Female physical aggression has been shown to lead to normalizing aggressive behavior and increasing the victimization of women (46). Thus, it may be possible to postulate that increasing female aggression during lockdown could lead to female victimization.

### Limitations of the Study

The main limitation of our study is the small sample size. Due to the lock-down conditions and the limited possibilities to involve a large number of women, we contacted the organizer of a fitness platform, obtaining a relatively small number of participants. To overcome this limitation, we employed non-parametric statistical tests. Additionally, because of the limitations of questionnaire opportunities, we could not survey the male partners of the surveyed participants. Thus, comparing women's responses to their spouses regarding aggressive behavior has not been conducted. Additionally, internet access can cause a bias toward a specific socio-economic section of the society, however, internet penetration in the Russian Federation was 81% in January 2020. Overall due to the exploratory nature of our studies, the interpretation of the results is only indicative of possible trends. Large-scale confirmatory studies are still needed to confirm our results (47).

## Conclusion

Our research shows a correlation between various states of emotions that active homemakers experience during lockdown conditions. We found that the entire considered cohort showed a high level of angriness and physical aggression. The feelings of uncertainty may have contributed to many participants reporting suffering from anxiety and angriness (48, 49). Notably, a significant association between anxiety and aggression score was found. Being affected by these negative emotions could lead to an increase in physical aggression by females. In turn, physical aggression could be correlated to the reported increase in the chances of female victimization during the lockdown. However, the exploratory nature of our study does not allow generalization on the whole of the Russian female population. Large-scale studies are still needed to confirm our findings.

## Data Availability Statement

The original contributions presented in the study are included in the article/supplementary material, further inquiries can be directed to the corresponding authors.

## Ethics Statement

Ethical review and approval was not required for the study on human participants in accordance with the local legislation and institutional requirements. However, ethical approval was obtained by the HAH (Institute of Scientific Personnel Training of the National Academy of Sciences). The patients/participants provided their written informed consent to participate in this study.

## Author Contributions

AK-M done the survey helped in concept. NK analysis and helped in writing. EM done statistical analysis and wrote parts of the manuscript. MD performed the analysis and helped in writing. ML and BW performed the analysis. MS supervised and wrote the manuscript. M-EM concept formation, wrote the manuscript and analysis. All authors contributed to the article and approved the submitted version.

## Conflict of Interest

The authors declare that the research was conducted in the absence of any commercial or financial relationships that could be construed as a potential conflict of interest.

## Publisher's Note

All claims expressed in this article are solely those of the authors and do not necessarily represent those of their affiliated organizations, or those of the publisher, the editors and the reviewers. Any product that may be evaluated in this article, or claim that may be made by its manufacturer, is not guaranteed or endorsed by the publisher.
